# Waiting Impulsivity: The Influence of Acute Methylphenidate and Feedback

**DOI:** 10.1093/ijnp/pyv074

**Published:** 2015-06-30

**Authors:** Valerie Voon, Yee Chien Chang-Webb, Laurel S. Morris, Ella Cooper, Arjun Sethi, Kwangyeol Baek, Jon Grant, Trevor W. Robbins, Neil A Harrison

**Affiliations:** Department of Psychiatry, University of Cambridge, Addenbrooke’s Hospital, Cambridge, United Kingdom (Dr Voon, Ms Chang-Webb, Ms Morris, Ms Cooper, Mr Sethi, Dr Baek, Dr Robbins, and Dr Harrison); Behavioral and Clinical Neuroscience Institute, University of Cambridge, Cambridge, United Kingdom (Dr Voon, Ms Morris, and Dr Robbins); Cambridgeshire and Peterborough NHS Foundation Trust, Cambridge, United Kingdom (Dr Voon); Department of Psychology, University of Cambridge, Cambridge, United Kingdom (Ms Morris and Dr Robbins); Department of Psychiatry, Brighton and Sussex Medical School, Brighton, United Kingdom (Ms Cooper, Mr Sethi, and Dr Harrison); Department of Psychiatry & Behavioral Neuroscience, University of Chicago, Chicago, IL (Dr Grant); Sackler Centre for Consciousness Science, University of Sussex, Brighton, United Kingdom (Dr Harrison); Sussex Partnership NHS Trust, Brighton, United Kingdom (Dr Harrison).

**Keywords:** addiction, binge drinking, impulsivity, methylphenidate, premature responding, stimulant dependence

## Abstract

**Background:**

The ability to wait and to weigh evidence is critical to behavioral regulation. These behaviors are known as waiting and reflection impulsivity. In Study 1, we examined the effects of methylphenidate, a dopamine and norepinephrine reuptake inhibitor, on waiting and reflection impulsivity in healthy young individuals. In study 2, we assessed the role of learning from feedback in disorders of addiction.

**Methods:**

We used the recently developed 4-Choice Serial Reaction Time task and the Beads task. Twenty-eight healthy volunteers were tested twice in a randomized, double-blind, placebo-controlled cross-over trial with 20mg methylphenidate. In the second study, we analyzed premature responses as a function of prior feedback in disorders of addiction.

**Results:**

Study 1: Methylphenidate was associated with greater waiting impulsivity to a cue predicting reward along with faster responding to target onset without a generalized effect on reaction time or attention. Methylphenidate influenced reflection impulsivity based on baseline impulsivity. Study 2: More premature responses occurred after premature responses in stimulant-dependent subjects.

**Conclusions:**

We show that methylphenidate has dissociable effects on waiting and reflection impulsivity. Chronic stimulant exposure impairs learning from prior premature responses, suggesting a failure to learn that premature responding is suboptimal. These findings provide a greater mechanistic understanding of waiting impulsivity.

## Introduction

The capacity to wait prior to responding and to weigh evidence prior to a decision are critical elements of behavioral regulation. These are subtypes of impulsivity known as waiting (Dalley et al., 2011) and reflection impulsivity (Kagan, 1966), respectively. Impulsivity is heterogeneous, with differing subtypes associated with distinct yet overlapping neural substrates.

Waiting impulsivity or premature responding describes anticipatory responses made prior to a cue predicting reward. It has been extensively investigated in rodent studies using the 5-Choice Serial Reaction Time task (5-CSRT) (Robbins, 2002) and shown to be both a predictor of compulsive substance use as well as a consequence of drug exposure. The neurochemistry underlying waiting impulsivity in rodents implicates dopaminergic, noradrenergic, and serotonergic mechanisms (Bari and Robbins, 2013). In rodents, methylphenidate (MPH) increases premature responding, an effect mediated by the beta-adrenergic receptor and D4 receptor (Milstein et al., 2010), and may be influenced by dose (Navarra et al., 2008) and baseline impulsivity (Tomlinson et al., 2014). MPH infusion into the rodent nucleus accumbens core also enhances premature responding (Economidou et al., 2012). Translational versions of tasks assessing waiting impulsivity have recently been developed in humans. These include the 4-Choice Serial Reaction Time task (4-CSRT), which maintains fidelity to the rodent 5-CSRT (Voon, 2014; Voon et al., 2014), and the Sussex-5-CSRT task (Sanchez-Roige et al., 2014). Premature responding has been shown to be elevated in methamphetamine, alcohol use disorders (AUDs), current smokers, and cannabis users with the 4-CSRT (Voon et al., 2014) and in binge drinkers (BDs) with the Sussex-5-CSRT task (Sanchez-Roige et al., 2014). Potential mechanisms contributing to waiting impulsivity include the role of motivational processes, proactive or tonic inhibition, timing deficits, and sensitivity to negative feedback and delay (Voon, 2014). In rodent studies, impulsive responses followed more frequently after errors that resulted in reward omission with excitotoxic lesions of the nucleus accumbens core (Christakou et al., 2004).

Reflection impulsivity describes the accumulation of evidence prior to decision (Kagan, 1966). In the Beads Task, participants view 2 jars with fixed probabilities of opposing ratios of red and blue beads. Beads are selected from 1 of the jars and shown to the participants. Participants must make a decision from which jar the beads are selected based on viewing the colored beads. The Beads task assesses reflection impulsivity in the probabilistic domain; participants are aware of the explicit probabilities of the alternate options with each piece of evidence accumulated associated with an expected probability (or level of certainty) of being correct. Using this task, elevated probabilistic reflection impulsivity has been observed in substance use disorders, pathological gamblers (Djamshidian et al., 2012), BDs (Banca et al., 2015), and patients with Parkinson’s disease with medication-induced behavioral addictions (Djamshidian et al., 2012). Reflection impulsivity tested using the Beads task is enhanced by dopamine receptor agonists though not by Levodopa (Djamshidian et al., 2013) in studies of Parkinson’s disease.

In the first study, we focused on MPH, an indirect catecholamine agonist that is commonly used as a cognitive enhancer in healthy individuals estimated at approximately 4% in college-age students in the United States (Bogle and Smith, 2009). MPH is also commonly used for the management of attention deficit hyperactivity disorder; this current study focuses on its use in young adult healthy volunteers. Acute MPH in healthy volunteers appears to have multiple influences on enhancing cognition, including enhancing set shifting and memory consolidation (Linssen et al., 2012), working memory and planning (Elliott et al., 1997; Mehta et al., 2000b), and improves motor response inhibition (Nandam et al., 2011; Pauls et al., 2012; Costa et al., 2013; Farr et al., 2014). However, some effects may be detrimental; MPH increases risk-taking behavior in healthy volunteers (Campbell-Meiklejohn et al., 2012). This study thus addresses how MPH affects performance of 2 novel impulsivity tasks in healthy volunteers.

In the second study, we analyzed data from our previous studies using the 4-CSRT (Voon et al., 2014) in subjects with disorders linked with aberrant dopaminergic integrity in order to explore associations between more chronic dopaminergic changes and characteristics of premature responses. For example, abstinent methamphetamine dependent (Stim) have blunted striatal dopamine receptor availability (Volkow et al., 2001b) associated with impulsivity (Lee et al., 2009), and individuals with AUDs have reduced ventral striatal dopamine transmission (Martinez et al., 2005) associated with alcohol craving (Heinz et al., 2004). Changes in dopamine transmission in obese subjects remains unclear with reported reductions in striatal D2 receptor binding that are associated with BMI (Wang et al., 2001) as well as no difference in underlying Dopamine (DA) capacity (Davis et al., 2009) in obese with binge eating disorder (BED) but enhanced dopamine transmission at presentation of food stimulus in BED (Wang et al., 2011). We have also recently reported enhanced premature responding in BDs at elevated risk for the development of AUD (Morris et al., 2015). Here we extend an examination of the characteristic features of premature responses in these groups by specifically assessing the role of prior feedback. The negative reinforcement model suggests that negative reinforcers such as stress or anxiety may drive addiction processes (Koob, 2013). Whether this is relevant as an endophenotype or early or late in the addiction process remains to be established (Wise and Koob, 2014). We hypothesize that Stim- and AUD-dependent individuals would have enhanced premature responses following negative feedback but not in healthy volunteers exposed to acute MPH or BDs, suggesting a role for development of negative reinforcement in the later stage of the addiction process.

## Methods

In the first study, we recruited young healthy volunteers above the age of 18 years who were medication-free and without any history of psychiatric or medical disorders. Participants were tested twice in a double-blind, within-subject, randomized placebo-controlled study with a 1-week cross-over period. A total of 20mg of short-acting MPH was administered, following which participants sat quietly or completed questionnaires. Participants were then tested at 1 hour postadministration equivalent to peak dose. Subjects were tested on the National Adult Reading Test (Nelson, 1982) for Verbal IQ. Subjects completed the Beck Depression Inventory (Beck et al., 1961), State and Trait Anxiety Inventory (Spielberger CD, 1983), and UPPS Impulsive Behaviour Scale (UPPS-P) (Whiteside and Lynam, 2001) to assess for depression, anxiety, and impulsivity.

In the second study, we reanalyzed data from the 4-CSRT previously reported comparing Stim, AUD, obese subjects with and without BED (Voon et al., 2014), and BDs compared with healthy volunteers focusing on novel analyses to examine the influence of prior feedback on premature responding. The recruitment and diagnostic criteria and inclusion and exclusion criteria were previously reported (Voon et al., 2014). The study was approved by the University of Cambridge Research Ethics Committee.

### Choice Serial Reaction Time Task

**Figure 1.**
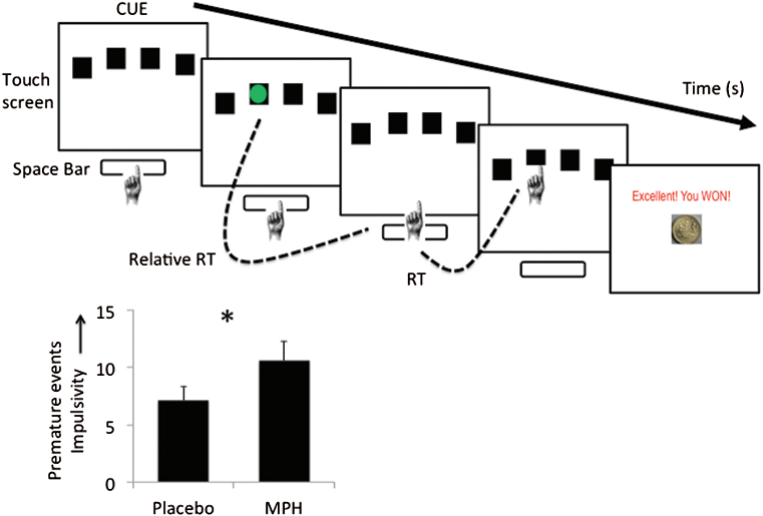
Premature responding task and outcomes. 4-Choice Serial Reaction Time task (4-CSRT). Reaction time (RT) was measured as the RT from green target onset to release of space bar; Movement time (MT) was measured as the RT from release of the space bar to touching the screen. The graph represents premature responses in healthy individuals on methylphenidate (MPH) or placebo. Higher premature responding represents greater impulsivity. Error bars represent between subject standard error of the mean. *P<.05

The 4-CSRT task (Figure 1) was developed based on the rodent 5-CSRT (Voon et al., 2014). Participants were seated in front of a 10.1 LCD touch screen monitor. When 4 boxes appeared on the screen, the participant pressed and held down the space bar on the keyboard with their dominant index finger, indicating the “cue onset” time. After a specified period (cue-target interval), a green circle target appeared briefly and randomly in 1 of the 4 boxes. Participants released the space bar and touched the box in which the target appeared. Baseline blocks without monetary feedback were used to individualize monetary feedback amounts for subsequent blocks based on the individual’s mean fastest reaction time (RT) and SD. The subsequent 4 Test blocks with monetary feedback were optimized to increase premature responding. This included variation of target duration, variability of the cue-target interval, and the presence of distractors. Accurate and timely responses were followed by individualized reward magnitude outcomes depending on the speed of responding. The task lasted 20 minutes and was programmed in Visual Basic with Visual Studio 2005. See Voon et al. (2014) for further task details.

The premature responding task consisted of 2 Baseline blocks and 4 Test blocks. Baseline blocks (level 3); Test block feedback (level 3); Very fast accurate responses (level 4); fast accurate responses (level 4); slow accurate responses (level 4); no response (level 4); premature response or incorrect responses (level 4); Test blocks (level 3).

#### Baseline Blocks

The baseline blocks were used to calculate the individual’s mean RT and SD to individualize feedback according to the individual’s RT and encourage individuals to respond faster. The first baseline block occurred at the start of the trial with the mean RT used for test block 1. The second baseline block occurred at the end of test block 1 with the mean RT from both baseline blocks used for test blocks 2 to 4. The subjects were told to respond as quickly as possible during the baseline blocks and the words “Keep going” appeared on the screen as feedback.

*Test block feedback*: Each baseline block had 20 trials, with the final 10 trials used to calculate mean RT and SD to individualize feedback and incentivize faster responding in subsequent test blocks. On test blocks, subjects saw both feedback (text and corresponding monetary image) and the cumulative total. The relationships between baseline block mean RT, SD, and test block feedback were as follows:

very fast accurate responses: For very fast accurate responses in which RT during a trial in the test blocks was < -0.5 SD of the baseline RT, the response was followed by the text “YOU WIN!! EXCELLENT!!” along with a £1 image. If subjects won £1 in 3 sequential trials, the feedback increased to £2.

fast accurate responses: For accurate responses in which test RT was between -0.5 SD and +0.5 SD of the baseline RT, the response was followed by the text “Very good. Keep going.” along with a 50-pence image. Test RTs that were accurate and between +0.5 SD and +1.5 SD of the baseline RT were followed by the text “Good. Keep going.” along with a 10-pence image.

slow accurate responses: Slow but accurate responses in which trial RTs were > +1.5 SD of the baseline RT were penalized and followed by the text “YOU LOSE!! TOO LATE!! HURRY UP!!” and an image of -£1 with a red X over the coin.

no response: If no responses were registered, the feedback was “TOO LATE!! GO FASTER!!” with an image -£1 with a red X.

premature response or incorrect responses: Neither premature responses (responding prior to target onset) nor incorrect responses (touching the incorrect box) were penalized. Following a premature response, subjects were required to touch the screen to complete the trial, which was followed by the text “Keep going.” An incorrect response was followed by the text ‘Keep going.” Thus, in both these cases, the response is suboptimal in that the time required for the trial is not rewarded and has parallels with a time out in the rodent literature.

#### Test Blocks

There were 4 test blocks with monetary feedback (40 trials/block). Subjects were instructed to respond as quickly as possible. They were told that they would earn money for their responses and would earn more money for faster responses. They were told that it was more important to be fast rather than accurate and that they would not lose money if they were inaccurate.

In the baseline blocks without feedback, the target duration was 64 milliseconds and the cue-target interval was 2 seconds. In test block 1 (long target) with monetary feedback, the target duration and cue-target interval were the same as the baseline blocks. In test block 2 (short target), the target duration was 32 milliseconds and the cue-target interval was 2 seconds. In test block 3 (variable interval), the target duration was 32 milliseconds and the cue-target interval varied from 2 to 10 seconds. In test block 4 (distractor), red circles followed by yellow circles appeared sequentially and randomly in 1 of the 4 boxes during the cue-target interval (2–10 seconds) prior to onset of the green target (target duration 32 milliseconds). The distractor circles were presented for 32 milliseconds for a random number. The distance between the touch screen and keyboard was held constant for each individual throughout the course of the experiment.

Primary outcome measures included total premature responses, which includes premature release (as reported in previous studies) (Voon et al., 2014; Worbe et al., 2014), and premature responses (touching the cue on the screen prior to target onset). Other secondary and exploratory outcome measures included motivation index ([baseline RT2 – baseline RT1]/baseline RT 1); RT (RT = time from target onset to space bar release); movement time (MT = time from release of the space bar to touch screen); accuracy (correct responses/correct responses + incorrect responses), where incorrect responses were trials in which the participant responded in time but to the wrong box (but were not penalized); late responses (during which participants were penalized by monetary loss); total won; and proportion of premature responses following a premature response, monetary win, or monetary loss (=premature responses following a premature response, monetary win, or monetary loss divided by total premature response, monetary win, or monetary loss, respectively).

### Beads Task

Participants were shown 2 jars on the computer screen with opposite ratios of red and blue beads (Jar 1: *P=.*80 red; *P=.*20 blue / Jar 2: *P=.*80 blue; *P=.*20 red) (Figure 3). They were informed of the bead ratio and were told that beads from 1 of the jars would be presented 1 at a time in the center of the screen. The participants’ goal was to infer whether the beads were drawn from Jar 1 or Jar 2. The participants were free to view as many beads as they wanted to a maximum of 20 beads before committing to their decision. The decision was followed by a confidence rating in which participants used a mouse to indicate the degree of confidence that their answer was correct on a line anchored at “not confident” to “very confident.” Participants were then informed that the next block would start. In this version, there was no feedback. The task controlled for working memory by showing the colored beads drawn across 2 rows at the top of screen. There was no time limit to the task. The primary outcome measure was the number of beads drawn prior to a decision or the amount of evidence accumulated. Secondary outcomes included subjective confidence and objective probability of the correct jar at the time of decision. There were 3 blocks of trials with the same bead order used in a previous study (Moutoussis et al., 2011).

### Statistics

All data were inspected for outliers (>3 SD from group mean) with outliers removed from analysis. The Kolmogorov-Smirnoff test was used to assess normality of distribution and log10 transformation applied to data that were not normally distributed. Paired *t* tests were used to assess data on MPH and placebo. The number of beads in the beads task was also analyzed as a function of baseline impulsivity by dividing groups based on a median split of high and low placebo baseline impulsivity or number of beads to decision (median = 7.33). The difference between the number of beads on placebo vs MPH was compared between the high and low baseline impulsivity groups using independent *t* tests. *P<.*05 was considered significant. For study 1, the ratio of premature responses following the highest positive feedback (+£2), negative feedback (-£1), or a premature response relative to total premature responses was compared using a paired *t* test for the comparison of MPH and placebo. For study 2, to allow comparisons between all the different groups, we combined the healthy controls and conducted a mixed-measures ANOVA with within-subject factor of feedback and between subject factor of group including healthy controls and all subject groups. *P<.*05 was considered significant.

## Results

In study 1, 28 participants were tested (female = 22; age 20.71 [1.84 SD] years; verbal IQ 113.41 [4.66 SD]; BDI 9.8 [10.2 SD]; STAI-state 40.5 [12.5 SD]; UPPS total 154.96 [18 SD]).

In study 2, 30 AUD, 30 obese with BED and 30 without BED, 23 Stim, and 32 BD were compared with all combined healthy volunteers (N=84). Primary diagnoses were confirmed by a psychiatrist using the DSM IV-TR criteria for substance dependence and Research Diagnostic Criteria for BED (Association, 2000). Healthy volunteers, AUD, and obese with and without BED were excluded if they had a current major depression or other major psychiatric disorder, including substance addiction (except nicotine), major medical illness, or taking psychotropic medications. Detailed subject characteristics have been previously reported (Voon et al., 2014). We have previously reported higher premature release scores (Voon et al., 2014). In Table 1, we report both premature release and total premature responses (premature response and release).

**Table 1.** Total Premature Responses and Premature Release

	Premature release (SD)	Total premature response (SD)
AUD	10.17 (8.79)	13.85 (10.03)
AUD-HV	6.02 (4.36)	8.03 (5.77)
T	2.317	2.755
P	0.024	0.008
Stim	13.35 (6.77)	18.76 (9.44)
Stim-HV	7.52 (5.59)	9.41 (6.68)
T	3.05	3.695
P	0.004	<0.001
BD	10.86 (7.21)	14.62 (7.74)
BD-HV	7.15 (6.12)	9.64 (8.11)
T	2.149	2.433
P	0.036	0.018
Methylphenidate-HV	8.41 (7.05)	10.63 (8.94)
Placebo-HV	5.96 (5.43)	7.07 (6.46)
	2.15	2.38
	0.053*	0.043*

*paired t-test.

Abbreviations: AUD, alcohol use disorder; HV, healthy volunteer; Stim, stimulant use disorder; BD, binge drinker.

### Effects of MPH on Waiting Impulsivity

As the MPH data for total premature responses, RT, MT, proportion of premature responses after a premature response, monetary win or monetary loss, accuracy, and late trials were not normally distributed, these data were log10 transformed. MPH was associated with significantly more total premature responses (nontransformed data reported in mean [SD]: placebo: 7.07 [6.46]; MPH: 10.63 [8.94], t_(28)_=-2.38, *P=.*043) with a trend for greater premature releases in the MPH compared with placebo condition (placebo: 5.96 [5.43]; MPH: 8.41 [7.05]; t_(28)_ =-2.15, *P=.*053).

**Figure 2.**
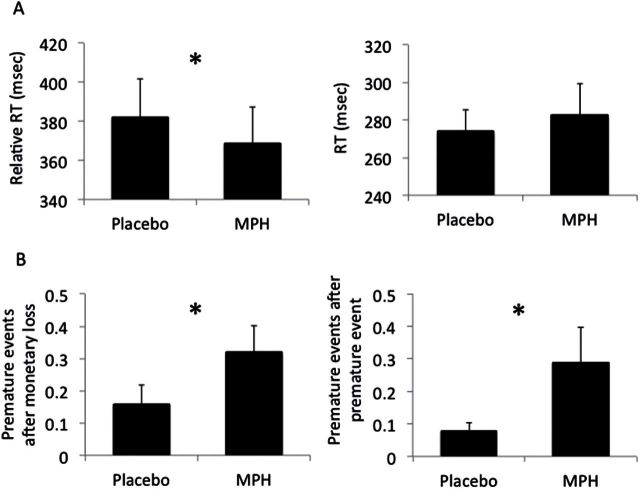
Secondary outcomes of premature responding task. (A) Reaction time (RT; left: RT = time from target onset to release of space bar) and movement time (right: MT = time from release of space bar to touching the screen) in healthy individuals on methylphenidate (MPH) or placebo. (B) Ratio of premature responses following monetary loss (left) and prior premature response (right). Error bars represent between subject standard error of the mean. *P<.05, ♮*P=.*065.

MPH was associated with faster RT (placebo: 381.91 [105.36] msec; MPH: 368.94 [95.85] msec, t_(28)_=2.16, *P=.*040). However, no differences were observed for MT (placebo: 274.31 [59.54]; MPH: 282.70 [87.36], t_(28)_=-0.46, *P=.*646) (Figure 2A).

There were no significant differences between MPH and placebo in the ratio of premature responses after a premature response (t=-1.25, *P=.*222), negative feedback (t=1.29, *P=.*210), or positive feedback (t=1.30, *P=.*206) (Table 2).

**Table 2.** Number of Premature Responses following Feedback

	Prem following prem	Prem following negative feedback	Prem following positive feedback
Healthy volunteers	1.96 (3.45)	0.86 (1.34)	4.69 (3.46)
	0.13 (0.14)	0.07 (0.12)	0.48 (0.25)
AUD	3.89 (6.77)	1.07 (1.38)	5.93 (5.49)
	0.18 (0.18)	0.09 (0.10)	0.51 (0.28)
Stim	5.09 (6.12)	0.76 (1.18)	7.94 (4.87)
	0.21 (0.16)	0.04 (0.05)	0.53 (0.24)
BD	3.62 (5.37)	0.86 (1.36)	6.31 (3.61)
	0.18 (0.18)	0.06 (0.08)	0.48 (0.20)
BED	0.70 (1.26)	0.55 (0.85)	3.96 (2.61)
	0.08 (0.13)	0.06 (0.09)	0.58 (0.29)
Obese	1.19 (2.10)	0.97 (1.20)	4.29 (3.65)
	0.10 (0.15)	0.12 (0.15)	0.47 (0.25)
Methylphenidate-HV	3.18 (1.00)	0.62 (0.57)	4.41 (4.25)
	0.17 (0.24)	0.04 (0.07)	0.52 (0.25)
Placebo-HV	1.0 (2.12)	0.57 (1.12)	4.25 (3.32)
	0.10 (0.14)	0.08 (0.14)	0.61 (0.24)

Reported as actual number of premature responses per category (mean (SD) and ratio per category relative to total premature responses (mean (SD).

There were no differences in motivational index (placebo: 0.22 [0.19]; MPH: 0.22 [0.16], t_(28)_=0.03, *P=.*975), accuracy (placebo: 0.92 [0.06], MPH: 0.92 [0.06], t_(28)_=-0.13, *P=.*897), late responses (placebo: 10.41 [11.74]; MPH: 7.81 [4.98], t=0.53, *P=.*601) or amount won (placebo: 1114.74 [428.83]; MPH: 1066.52 [405.64], t_(28)_= 0.49, *P=.*627).

The ratio of correct fast (win £2), correct slow (win £1 or £0.50), miss, or late (lose £1) responses following a premature response, monetary win, or monetary loss were also examined to assess specificity of the premature response findings. There were no significant differences between MPH and placebo in these measures (*P*>.05).

### Effects of MPH on Evidence Accumulation

**Figure 3.**
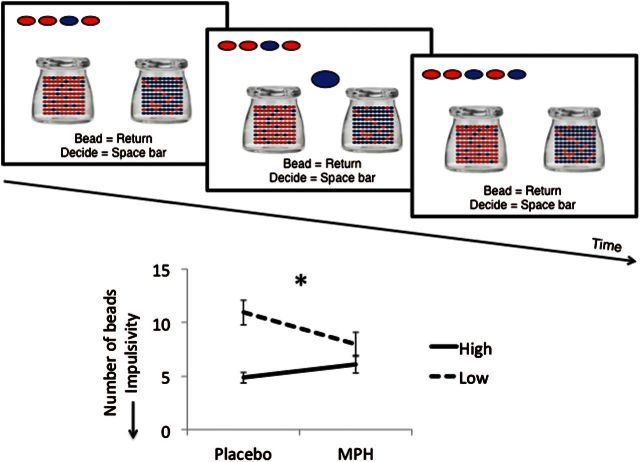
Reflection impulsivity task. Beads task. The graph represents the number of beads or evidence accumulated in healthy individuals on methylphenidate (MPH) or placebo as a function of high or low reflection impulsivity at baseline on placebo. Lower number of beads represents greater impulsivity. Error bars represent between subject standard error of the mean. *P<.001

As the number of beads and confidence were not normally distributed, the data were log10 transformed. One participant was removed from placebo number of beads and confidence and MPH confidence, as the data were outliers (>3 SD from group mean). MPH was not different from placebo in the number of beads to decision (placebo: 7.81 [4.07]; MPH 7.17 [3.37], t=1.35, *P=.*250). However, when analyzed as a function of baseline placebo impulsivity, participants with low baseline impulsivity (beads>7.33 at baseline) showed an increase in reflection impulsivity on MPH with an opposite (decrease) observed in those with high baseline impulsivity (beads<7.33 at baseline) (difference MPH – placebo in number of beads: high baseline impulsivity: 1.25 [2.04]; low baseline impulsivity: -2.55 [2.61], t=4.14, *P<.*001) (Figure 3). Similarly, baseline placebo impulsivity (number of beads) was negatively correlated with the difference between the number of beads on placebo and on MPH (Pearson correlation coefficient: R^2^=0.28, *P=.*003).

There were no differences between MPH and placebo on secondary outcome measures of subjective confidence (placebo: 438.40 [65.26]; MPH: 456.76 [62.89], t=-1.67, *P=.*108), objective probability, or levels of uncertainty at the time of decision (placebo: 0.91 [0.17]; MPH: 0.94 [0.12], *P=.*273).

There was no relationship between premature responses and number of beads or confidence under MPH or placebo (*P*>.05). The total premature responses at baseline were divided into high (9.25 [SD 9.74] and low 2.75 [SD 1.14]) and the influence of MPH (high impulsive: 12.83 [SD 9.34]; low impulsive: 12.08 [7.20]) assessed using mixed-measures ANOVA. There was a group effect (*P=.*021), a trend towards a medication effect (*P=.*052), and no interaction between group and medication (*P=.*117). A similar analysis of premature releases showed a group effect (*P=.*002) but no medication (*P=.*143) or interaction (*P=.*613) effect.

The relationship between the 2 forms of impulsivity was further investigated. First, we divided it into high and low impulsivity based on a median split from the beads task. We then assessed the relationship between total premature responses on placebo in those with high reflection impulsivity (total premature responses: 10.00 [SD 8.97]) and low reflection impulsivity (5.50 [SD 3.58]) (*P=.*123) on placebo. Although the difference was not significant, notably given the differences between the raw scores, this might suggest a potential relationship between the 2 measures given a sufficient sample size. Similarly, we investigated the correlation between total premature responses and premature responding (placebo: Pearson correlation: r=0.281; *P=.*156; MPH: r=-0.106; *P=.*590)

### Premature Responses following Feedback in Disorders of Addiction

There was a main effect of feedback (F(2,222) =210.78, *P*<.001) and a group by feedback interaction (F(10,446) =2.63, P = 0.004) and no group effect (F(5,223) =1.12, *P=.*353). To further understand the interaction effects, we conducted posthoc analyses using Tukey test. There were significant group differences following a premature response (*P=.*008) and negative feedback (*P=.*046) but not following positive feedback (*P=.*439). Stim subjects made more premature responses following a premature response compared with healthy volunteers (*P=.*019), BED (*P=.*001), and obese without BED (*P=.*005). BED subjects made fewer premature responses following a premature response compared with BD (*P=.*019) and AUD subjects (*P=.*027). Obese subjects made more premature responses following negative feedback compared with healthy volunteers (*P*=.048), Stim (*P=.*002), BED (*P=.*019), and BD subjects (*P=.*023).

## Discussion

Here we show a dissociation of the effects of MPH on 2 subtypes of impulsivity. Specifically, MPH enhanced waiting impulsivity or premature responding prior to a cue predicting reward across participants in healthy volunteers. In contrast, MPH influences on reflection impulsivity were dependent on baseline reflection impulsivity, with low baseline impulsivity participants showing an increase and high baseline participants a decrease in reflection impulsivity.

On MPH, participants responded earlier to the target onset (ie, faster relative RT from target onset to space bar release) without a generalized speeding of RT (ie, RT from space bar release to touching the screen) or attention (ie, accuracy or missed responses). Thus, MPH may enhance task-specific attentional salience (ie, to the target predicting reward). In participants with low baseline reflection impulsivity, MPH enhanced impulsivity with an opposing effect in participants with high baseline reflection impulsivity.

### Feedback Effects

**Figure 4.**
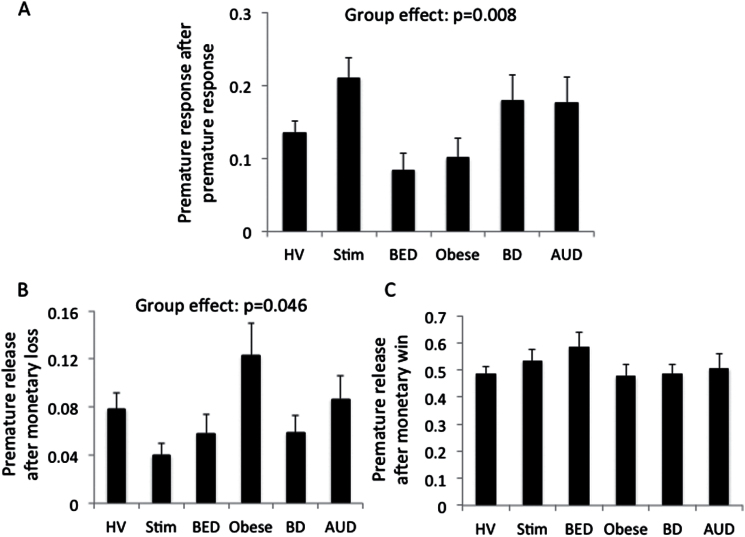
Premature response following feedback. (A) Ratio of premature responses following a premature response comparing healthy volunteers (HV), subjects abstinent from methamphetamine dependence (Stim) and alcohol use disorders (AUDs) and in binge drinkers (BDs) and obese subjects with and without binge eating disorder (BED). Ratio of premature responses following monetary loss (B) and following a monetary win (C).

We have previously shown that premature responding is elevated in Stim, AUD, and BD subjects but not in obese subjects with and without BED (Voon et al., 2014). Here we show group by feedback interaction differences in premature responding, particularly following a premature response and negative feedback (Figure 4). The influence of premature response was driven particularly by Stim subjects and BED: Stim subjects made more premature responses following a premature response compared with healthy volunteers and with both obese subjects with and without BED, whereas BED subjects made fewer premature responses following a premature response compared with BD and AUD subjects. These findings in Stim subjects of a repeat of premature response following a premature response suggest impaired learning from a prior premature response and an aberrant recognition that premature responses are suboptimal. This might suggest that the lack of a reward or the brief time delay following a premature response is insufficiently salient in Stim subjects.

Obese subjects made more premature responses following negative feedback compared with healthy volunteers, Stim, BED, and BD subjects, suggesting possibly enhanced frustrative motivation responding. These findings might suggest a role for enhanced sensitivity to negative feedback (Wise and Koob, 2014).

### Acute MPH Effects on Premature Responding

MPH acts by inhibiting dopamine and noradrenergic transporters and has been shown to increase presynaptic striatal dopamine (Volkow et al., 2001a) and striatal dopamine synthesis capacity (Henkel, 1981) as well as increasing synaptic levels of noradrenaline through its blockade of noradrenergic transporters (Hannestad et al., 2010) in humans. In rodents, MPH increases premature responding, an effect blocked by the beta-adrenergic receptor blocker propranolol and a D4 receptor antagonist but not by central noradrenergic depletion or D1 or D2 receptor antagonists (Milstein et al., 2010). These findings suggest that the influence of MPH on premature responding acts specifically via the beta-adrenergic receptor and D4 receptor. Infusion of MPH into the nucleus accumbens core but not the shell enhances premature responding, whereas infusion of atomoxetine, a specific noradrenergic reuptake inhibitor, into the nucleus accumbens shell but not the core decreases premature responding (Economidou et al., 2012). This indicates neurochemical and anatomical dissociability in the nucleus accumbens on premature responding. The influence of MPH on premature responding may also be related to dose as MPH improves attention (percent correct) in the 5CSRT across multiple doses, but only the highest dose of MPH increased premature responding (Navarra et al., 2008). Atomoxetine consistently decreased premature responding and, less consistently, improved attention in rodents on both the 5-CSRT (Robinson et al., 2008; Baarendse and Vanderschuren, 2012; Fernando et al., 2012; Paterson et al., 2012) and in a rodent gambling task (Baarendse et al., 2013). Thus, our finding of elevated premature responses following MPH in healthy adults converges with the rodent literature whereas atomoxetine may be more likely to decrease premature behavior.

In healthy humans, MPH improves motor inhibition as measured by stop signal task performance, possibly by enhancing salience of the stop signal and increasing attentional capture (Nandam et al., 2011; Pauls et al., 2012; Costa et al., 2013; Farr et al., 2014). Similarly, the influence of MPH on premature responding in this study may be related to enhanced salience of the target, hence hastening RT to the target or enhanced salience of negative feedback and therefore increasing premature responses following negative feedback. Importantly, we show enhanced RT to the target but no overall effect on RT, suggesting a specific mechanism involving the target or negative feedback. In an fMRI study of MPH in healthy volunteers using a 4-CSRT task that did not focus on premature responding, MPH was associated with enhanced activity in attention and preparatory motor regions (Muller et al., 2005). An influence of task-difficulty and plasma levels was observed with a positive correlation between greater area under the plasma MPH-time curve and activity of attentional and motor preparatory regions only in the condition of greater task difficulty (Muller et al., 2005). In healthy volunteers, MPH increases activity in a dorsal attentional network, including the parietal and prefrontal cortex (Tomasi et al., 2011). Overall these findings suggest the influence of MPH on premature responding may relate to enhanced task-dependent attentional salience and motor preparation.

Acute MPH in healthy volunteers appears to have multiple influences on enhancing cognition, including enhancing set shifting and memory consolidation (Linssen et al., 2012) as well as working memory and planning. Improvements in working memory were related to decreases in cerebral blood flow in lateral prefrontal and parietal cortices (Elliott et al., 1997; Mehta et al., 2000b) and also to baseline-dependent effects (Mehta et al., 2000a, 2004). However, not all effects are necessarily positive. MPH increases risk-taking behavior in healthy individuals (Campbell-Meiklejohn et al., 2012). In the current study, we show that acute MPH increases premature responding along with causing an enhanced likelihood of a premature response following a prior premature response. This suggests that MPH may also impair the capacity to learn that premature responses are suboptimal responses, possibly by a relative decrease in attentional salience. However, we also note that there were no overall differences in the amount of money won, suggesting that MPH did not impair optimal task performance.

### Acute MPH Effects on Evidence Accumulation

We also show that MPH influences evidence accumulation prior to a decision in a manner that is dependent on baseline impulsivity. These divergent effects of MPH were previously demonstrated for other forms of impulsivity. For example, MPH and atomoxetine were both shown to decrease premature responding in high-impulsive rodents in one study (Tomlinson et al., 2014). MPH also enhances motor inhibition as measured using the stop signal task, reducing the stop signal reaction time (SSRT) in slow-responding rodents but increasing SSRT in fast responders (Eagle et al., 2007). In humans, individuals with higher baseline impulsivity such as those with attention deficit hyperactivity disorder may respond differently to MPH (Caprioli et al., 2015). Overall, this suggests an influence of baseline on medication effects, thus emphasizing potential differences between medication effects in healthy volunteers and pathological groups with elevated impulsivity. In the current study, there were no differences in levels of uncertainty or objective probability of the correct choice at the time of decision or in subjective confidence, suggesting that the effects were indeed related to reflection impulsivity. In line with the current finding of reduced reflection impulsivity in high-impulsive participants, greater reflection impulsivity as tested using the Matching Familiar Figures Test in individuals with attention deficit hyperactivity disorder is decreased following MPH (Brown and Sleator, 1979). However, there were no differences in reflection impulsivity comparing ADHD and healthy volunteers as measured using the Information Sampling Task along with a lack of an influence of MPH (DeVito et al., 2009). We have previously shown that BDs were impaired on the beads task but not the IST (Banca et al., 2015), a dissociation similarly shown in studies in schizophrenia (Fine et al., 2007; Moutoussis et al., 2011) (Huddy et al., 2013) suggesting underlying task differences. We argue that the beads task, unlike the IST, may be more likely to increase impulsivity, as the task is less visually explicit and requires participants to be reliant on their own internal task representation and future outcomes (Banca et al., 2015).

### Limitations

This current study would benefit from assessment under differing doses at 20 and 40mg to assess dose effects. Furthermore, whereas MPH appears to improve motor response inhibition in healthy volunteers with a possible enhanced effect in ADHD, the role of premature responding in ADHD and the influence of chronic MPH remains to be tested. We also note that baseline scores of premature responding may subtly differ, possibly as a result of differences in the touch screen utilized. In the study of pathological groups (study 2), a larger 12.1-inch Paceblade touchscreen was used, whereas a 10.1-inch touch screen was used in Study 1, which may contribute to differences in baseline responses. We further note that subjects with lower beads accumulated (greater reflection impulsivity) had higher raw scores of total premature responses (greater waiting impulsivity) with the opposite observed in those with lower reflection impulsivity. Although there were no significant differences either with a correlation analysis or a median split, a larger sample size might reveal a relationship between the 2 measures. Thus, those who accumulate less evidence may also have difficulties with anticipatory responding.

## Conclusions

Our findings are immediately relevant to young healthy volunteers without a history of ADHD. While acute MPH may have positive effects as a cognitive enhancer in healthy volunteers, MPH may also enhance specific forms of impulsivity, including the capacity to wait before acting. Our findings suggest that chronic exposure to stimulants is associated with a failure to learn that premature responses are suboptimal. These findings add to a mechanistic understanding of waiting impulsivity and possible means of therapeutic interventions.

## Statement of Interest

V.V. and N.A.H. are Wellcome Trust (WT) intermediate Clinical Fellows. L.S.M. is an MRC student. The BCNI is supported by a WT and MRC grant. The authors report no conflicts of interest. T.W.R. consults for Cambridge Cognition, Lundbeck, Teva, Shire Pharmaceuticals, and Otsuka, has research grants from Lundbeck, GSK, Royalties Cambridge Cognition, and receives editorial honoraria from Springer, Elsevier.
